# Electron–Phonon Coupling and Phonon Dynamics in Single-Layer NbSe_2_ on Graphene: The Role of Moiré Phonons

**DOI:** 10.1021/acsnano.4c16399

**Published:** 2025-02-26

**Authors:** Amjad Al Taleb, Wen Wan, Giorgio Benedek, Miguel M. Ugeda, Daniel Farías

**Affiliations:** † Departamento de Física de la Materia Condensada, Universidad Autónoma de Madrid, 28049 Madrid, Spain; ‡ 226245 Donostia International Physics Center, Paseo Manuel de Lardizábal 4, 20018 San Sebastián, Spain; § Dipartimento di Scienza dei Materiali, Università di Milano-Bicocca, 20125 Milano, Italy; ∥ Ikerbasque, Basque Foundation for Science, 48013 Bilbao, Spain; ⊥ Centro de Física de Materiales, Paseo Manuel de Lardizábal 5, 20018 San Sebastián, Spain; # Instituto Nicolás Cabrera, Universidad Autónoma de Madrid, 28049 Madrid, Spain; ∇ Condensed Matter Physics Center (IFIMAC), Universidad Autónoma de Madrid, 28049 Madrid, Spain

**Keywords:** electron−phonon coupling, phonons, NbSe_2_, graphene, superconductor, Moiré structure

## Abstract

The interplay between substrate interactions and electron–phonon coupling in two-dimensional (2D) materials presents a significant challenge in understanding and controlling their electronic properties. Here, we present a comparative study of the structural characteristics, phonon dynamics, and electron–phonon interactions in bulk and monolayer NbSe_2_ on epitaxial bilayer graphene (BLG) using helium atom scattering (HAS). High-resolution helium diffraction reveals a (9 × 9)­0° superstructure within the NbSe_2_ monolayer, commensurate with the BLG lattice, while out-of-plane HAS diffraction spectra indicate a low-corrugated (3√3 × 3√3)­30° substructure. By monitoring the thermal attenuation of the specular peak across a temperature range of 100 to 300 K, we determined the electron–phonon coupling constant (λ_HAS_) as 0.76 for bulk 2H-NbSe_2_. In contrast, the NbSe_2_ monolayer on graphene exhibits a reduced λ_HAS_ of 0.55, corresponding to a superconducting critical temperature (*T*_C_) of 1.56 K according to the MacMillan formula, consistent with transport measurement findings. Inelastic HAS data provide, besides a set of dispersion curves of acoustic and lower optical phonons, a soft, dispersionless branch of phonons at 1.7 meV, attributed to the interface localized defects distributed with the superstructure period, thus termed Moiré phonons. Our data show that Moiré phonons contribute significantly to the electron–phonon coupling in monolayer NbSe_2_. These results highlight the crucial role of the BLG in the electron–phonon coupling in monolayer NbSe_2_, attributed to enhanced charge transfer effects, providing valuable insights into substrate-dependent electronic interactions in 2D superconductors.

## Graphical abstract

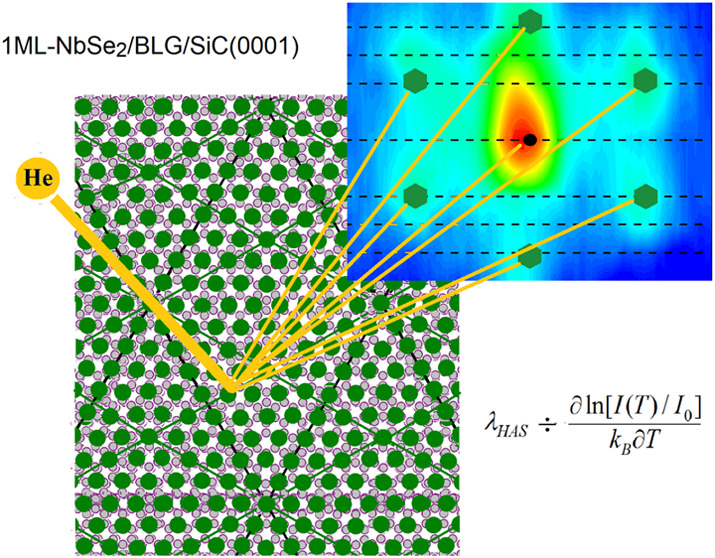

## 1 Introduction

Layered transition metal chalcogenides (TMD) are well-suited systems to study the effects of dimensionality and thickness on collective electronic phases like superconductivity and charge density waves (CDW). These are generally manifestations of electron–phonon interactions, leading to electron–electron (hole–hole) pairing and electron–hole ordering, respectively. Bulk 2H-NbSe_2_ is especially interesting due to the coexistence of superconducting and CDW phases: a CDW with a (3 × 3) periodicity is known to set in below 33 K,^1^ while superconductivity sets in at *T*_c_ = 7.2 K.^2^ In the monolayer NbSe_2_ limit, different results have been reported depending on the supporting substrate. On epitaxial bilayer graphene (BLG) on SiC(0001), *T*_C_ is depressed to 1.5 K whereas the critical temperature for the appearance of the CDW remains unchanged (33 K).^3^ However, when the monolayer is held on sapphire, superconductivity was observed to set in at *T*_c_ = 3 K, while the CDW critical temperature increases to 145 K.^4^ These results were interpreted in terms of an increase in the electron–phonon coupling in single-layer NbSe_2_/sapphire. Regarding charge transfer, there is little difference between sapphire (work function ∼4.5 eV^5^) and BLG/SiC (work function 4.30 eV^6^), when compared to the NbSe_2_ work function of 5.9 eV.^7^ Thus, these different behaviors in the presence of similar charge transfer raise the question about the possible role of BLG underneath in both superconductivity and CDW and more specifically, on the strength of the electron–phonon coupling in single-layer NbSe_2_.

In this work, we report measurements of the electron–phonon coupling constant (mass-enhancement factor) λ_HAS_ in single-layer NbSe_2_/BLG/SiC­(0001) by high-resolution helium atom scattering (HAS) spectroscopy,^8^ which enabled us to extract information about the surface structure on a long-period (nm) scale and related low-energy (meV) dynamics. Our He-diffraction results show that single-layer NbSe_2_ on BLG develops a commensurate superstructure, where a (9 × 9) NbSe_2_ monolayer supercell matches with a small tensile strain a (13 × 13) BLG supercell. A comparatively intense, dispersionless low-energy (1.7 meV) phonon branch is also observed. This effect induced by the superstructure on phonon dynamics is analogous to what observed in the electronic structure, like the occurrence of ultraflat electronic bands, e.g., in twisted bilayers of WSe_2_ ^9^ and of graphene,^10,11^ ultimately responsible for the observed superconductivity in these heterostructures.

A recent quantum-theoretical approach showed how the thermal attenuation of the HAS specular peak from metal surfaces, described by the Debye–Waller (DW) exponent, can directly provide the electron–phonon coupling constant (mass-enhancement factor λ, here denoted λ_HAS_ when obtained with this method)^12^ This method to derive λ_HAS_ has been recently extended to other classes of conducting surfaces, like those of layered TMDs.^13,14^ and supported graphene monolayers,^15^ and is here applied to the present NbSe_2_ heterostructures to elucidate the role of the low-energy phonon branch in the electron–phonon coupling. In general, HAS spectroscopy should be relevant for the characterization of twisted layered heterostructures and their functional properties in the new area of *twistronics*.^16^

## 2 Results and Discussion

### 2.1 Diffraction

Monolayers of NbSe_2_ were grown on BLG on 6H-SiC(0001) by molecular beam epitaxy in the homemade UHV-MBE system at the DIPC in San Sebastián. Samples with a coverage of 2–3 layers of NbSe_2_ have also been prepared. Before being shipped to Madrid for HAS measurements, the samples were capped with a ∼10 nm film of Se (see Supporting Information for more details on sample preparation). Figure 1a shows the HAS angular distribution measured for single-layer NbSe_2_/BLG/SiC­(0001) (red curve) along the (1000) direction, and is compared to that for a 2–3 layers of NbSe_2_/BLG/SiC­(0001) (green curve) and for the bulk NbSe_2_(0001) surface (black curve), all recorded at *T* = 100 K. Note that these data were recorded with a HAS system that allows the detector to rotate 200° in the scattering plane while keeping the angle of incidence fixed.^17^ The spectrum for bulk NbSe_2_ shows numerous intense diffraction peaks, indicating a highly corrugated surface, similar to those typically found on semiconductor surfaces. A comparable diffraction spectrum is observed for the few-layer sample, while the monolayer NbSe_2_ exhibits a significantly different pattern. In this case, the diffraction intensities are much weaker than the specular scattering, suggesting a metallic surface for the monolayer due to significant charge transfer from the substrate. This is consistent with the comparatively large difference between the work function of bulk NbSe_2_ (5.9 eV) [7] and that of few-layer NbSe_2_/BLG (4.30 eV).^6^

**Figure 1:**
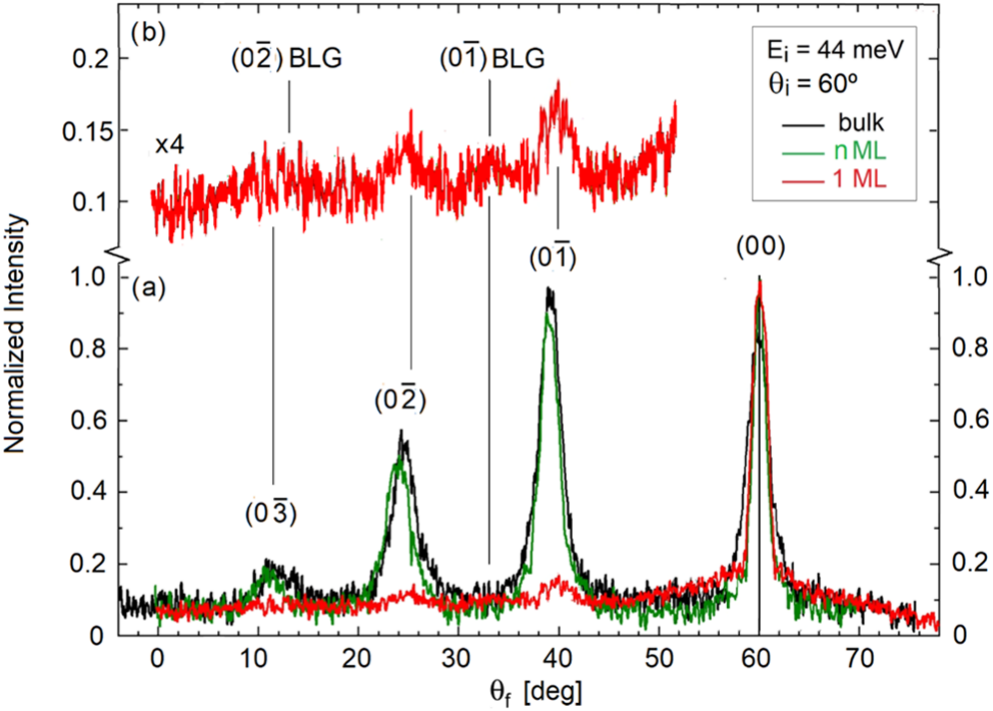
(a) Angular distribution of HAS along the (1000) direction (ΓM) of the surfaces of bulk NbSe_2_(0001) (black curve), few-layer NbSe_2_/BLG/SiC­(0001) (green curve) and single-layer NbSe_2_/BLG/SiC­(0001) (red curve). The angle of incidence is 60° and the incident energy of the He beam is *E*_*i*_ = 44 meV. (b) A factor-4 expansion of the diffraction spectrum for the monolayer NbSe_2_, where the BLG (01̅) diffraction peak is also visible, as an effect of the charge transfer.

The charge transfer is also responsible of a small shift of the diffraction peaks of the NbSe_2_ monolayer as compared to those of the bulk and few-layer samples. For the (01̅) diffraction the latter occur at θ_*f*_ = 39.5°, which gives an in-plane lattice constant *a*_N_ = 3.44 Å, in perfect agreement with the value measured in NbSe_2_ multilayer flakes,^18^ while the (01̅) peak maximum for the monolayer is shifted to 39.9°. This corresponds to *a*_N_ = 3.52 Å, i.e., to *a* ∼ 2.3% expansion of the in-plane monolayer lattice constant. The electronic charge transfer leaves behind a positive potential in the substrate and, therefore, a component in the surface charge density distribution reflecting the substrate periodicity. Actually, the ×4 magnification of the diffraction in the monolayer (Figure 1b) hints to a maximum around θ_*f*_ = 33°, where the BLG (01̅) diffraction peak is expected to occur for the known graphene lattice parameter of 2.46 Å.^19,20^

The observation of the diffraction peaks of both the monolayer NbSe_2_ and the BLG substrate induced by a large charge transfer is confirmed by the high-resolution HAS diffraction pattern displayed in Figure 2. This spectrum was measured using the HAS instrument with a time-of-flight (TOF) arm and a fixed angle of 108° between the incoming and outgoing beams. In this setup, angular distributions are measured by rotating the crystal around an axis perpendicular to a plane defined by the incoming beam and the normal to the sample surface.^21^ Throughout this paper we will use the term rocking angle (φ) which means the scattering angle relative to the specular position. The (01̅) BLG diffraction peak at φ = −22.1° is about one-half in size as that of single-layer NbSe_2_ at φ = −15.2° and comparable in sharpness, and corresponds to the in-plane lattice constants *a*_BLG_ = 2.47 ± 0.02 Å and *a*_N_ = 3.55 ± 0.02 Å. This value of *a*_N_ confirms the in-plane expansion (here 3.2 ± 0.6%) of the NbSe_2_ monolayer with respect to the bulk induced by the charge transfer.

**Figure 2:**
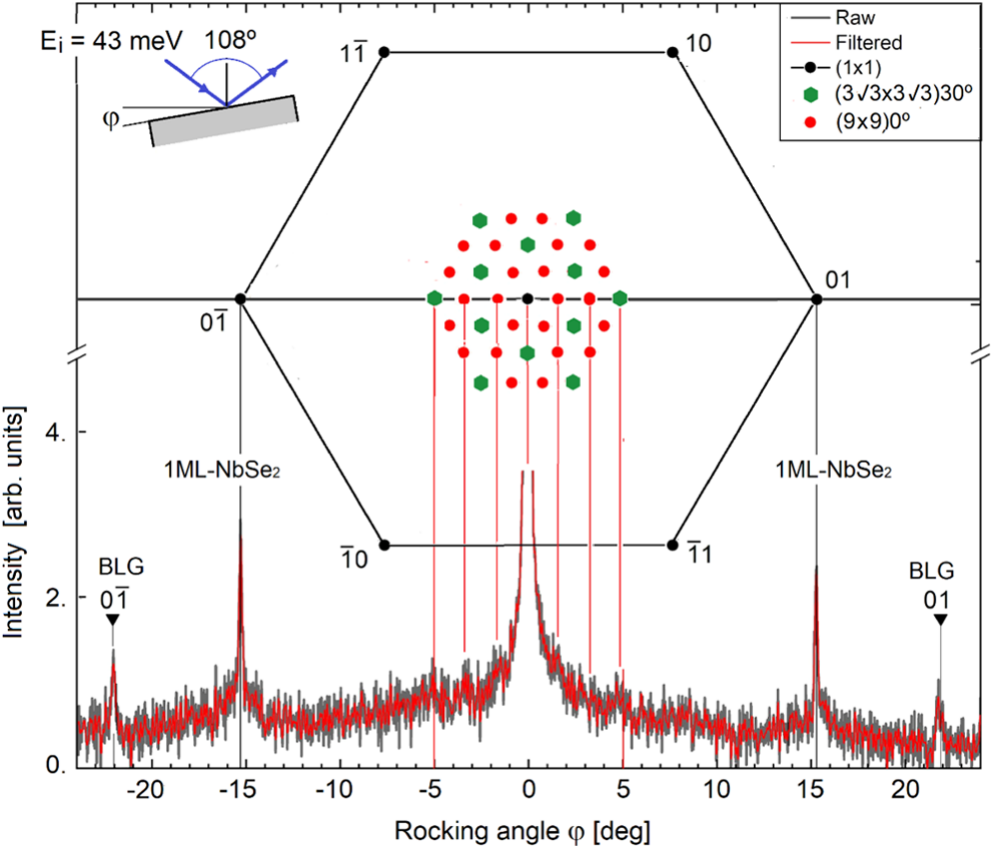
High-resolution HAS angular distribution measured as a function of the rocking angle (top-left inset) along the (1000) direction (ΓM) of single-layer NbSe_2_/BLG/SiC­(0001) at *T* = 100 K, with a fixed scattering angle of 108° and incident energy of 43 meV. The upper part of the figure shows the corresponding diffraction spots in the reciprocal space of single-layer NbSe_2_ (black dots) and those near the specular peak of the single-layer NbSe_2_/BLG superstructures indicated in the top-right inset and discussed in the text.

Since the diffraction components of both the single-layer NbSe_2_ and BLG are observed in Figure 2, the small features at φ ≅ −5, −3.3, −1.7 and 5° near the specular peak and discernible above the noise (especially in the filtered data, red line), are assigned to a commensurate superstructure with a unit cell aligned with those of the two component lattices. The lattice constant ratio *a*_N_/*a*_BLG_ = 1.44 is very close to 13/9 = 1.4̅, suggesting that the hexagonal superlattice unit cell is formed by the coincidence of a (9 × 9)­0° cell of the NbSe_2_ monolayer with a (13 × 13)­0° cell of BLG. This is illustrated by Figure 3a, which shows the interface Se ions (green atoms) positioned over the graphene (gray atoms), with the superlattice unit cell edges marked in red. The corresponding theoretical diffraction spots around the origin (specular peak) in the reciprocal plane are shown in the upper part of Figure 2 (red and green dots), with some spots closely corresponding to the small features described above.

**Figure 3:**
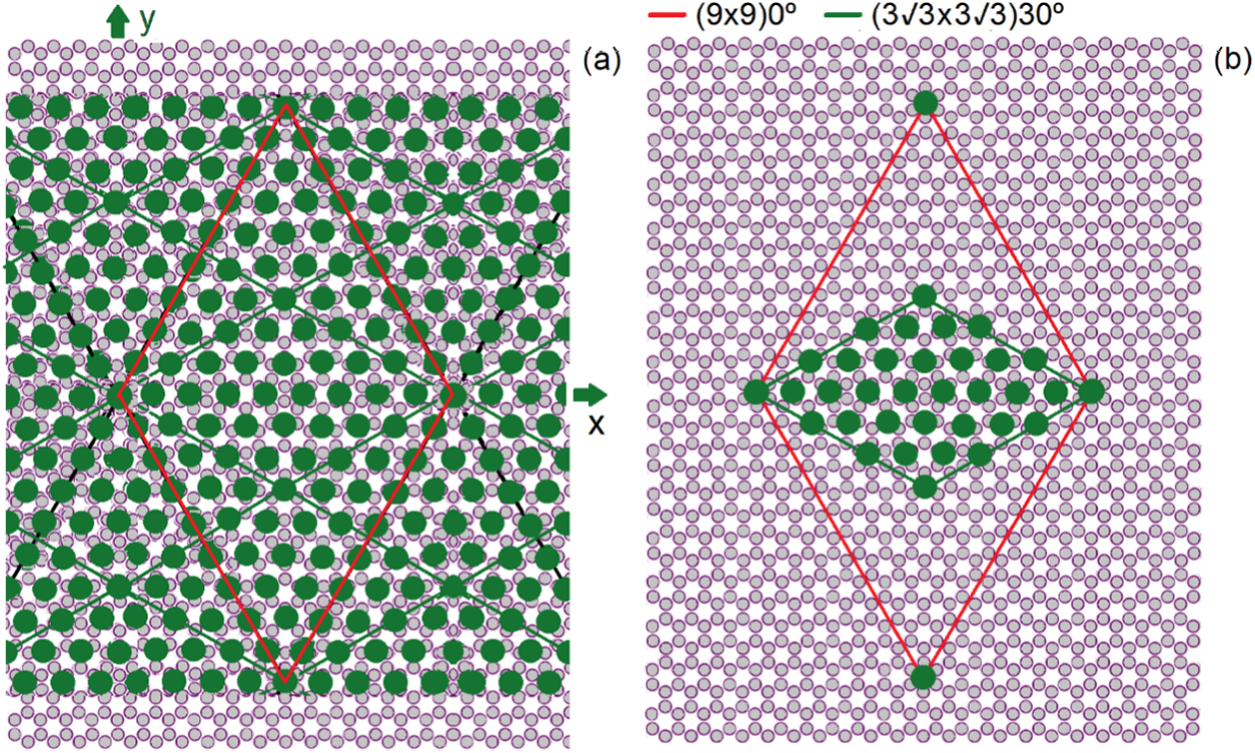
(a) Structure of the single-layer NbSe_2_/BLG interface showing the Se ions (green full circles) on graphene (gray C atoms). The red lines show the unit cell of the single-layer NbSe_2_ (9 × 9)­0° supercell matching the (13 × 13)­0° supercell of BLG. (b) The reduced (3√3 × 3√3)­30° unit cell of single-layer NbSe_2_ (green contour line) in case of equivalence between the BLG high-symmetry hexagonal sites (left and right corners of the reduced cell) and the trigonal symmetry sites (top and bottom corners), approximately produced by the addition of the second BLG graphene layer (not shown).

The presence of a second graphene layer underneath causes the Se ions at the hexagonal symmetry sites (e.g., the corners of the (9 × 9)­0° unit cell in Figure 3a) to be almost equivalent, in terms of adsorption energy, to those at the trigonal symmetry sites (e.g., the top and bottom atoms of the reduced (3√3 × 3√3)­30° unit cell, as shown in Figure 3b). If these sites were strictly equivalent, the actual (3√3 × 3√3)­30° supercell would be three times smaller, and only the diffraction peaks corresponding to the green spots in Figure 2 would appear. However, even with approximate equivalence, the green spots—especially the first six, which form a 30° rotated hexagon in Figure 2—are reinforced.

Out-of-plane measurements made using the HAS confirm this effect. The HAS intensity, mapped as a function of the final angle θ_*f*_ and out-of-plane azimuth angle ϕ_*f*_ for a fixed incident energy *E*_*i*_ = 56 meV and incident angle θ_*i*_ = 60° (see Figure 4b; kinematics explained in Figure 4a), shows maxima at the expected positions for the (3√3 × 3√3)­30° supercell (green hexagons). Note that the hexagonal pattern is slightly rotated clockwise relative to the calculated maxima positions, due to a minor azimuthal misalignment of the NbSe_2_ lattice with respect to the sagittal plane ϕ_*i*_ = 0° (see Figure SI-1 for details). However, this small instrumental misalignment does not undermine the strong evidence supporting the (3√3 × 3√3)­30° supercell as the approximate commensurate structure for single-layer NbSe_2_/BLG/SiC­(0001).

**Figure 4:**
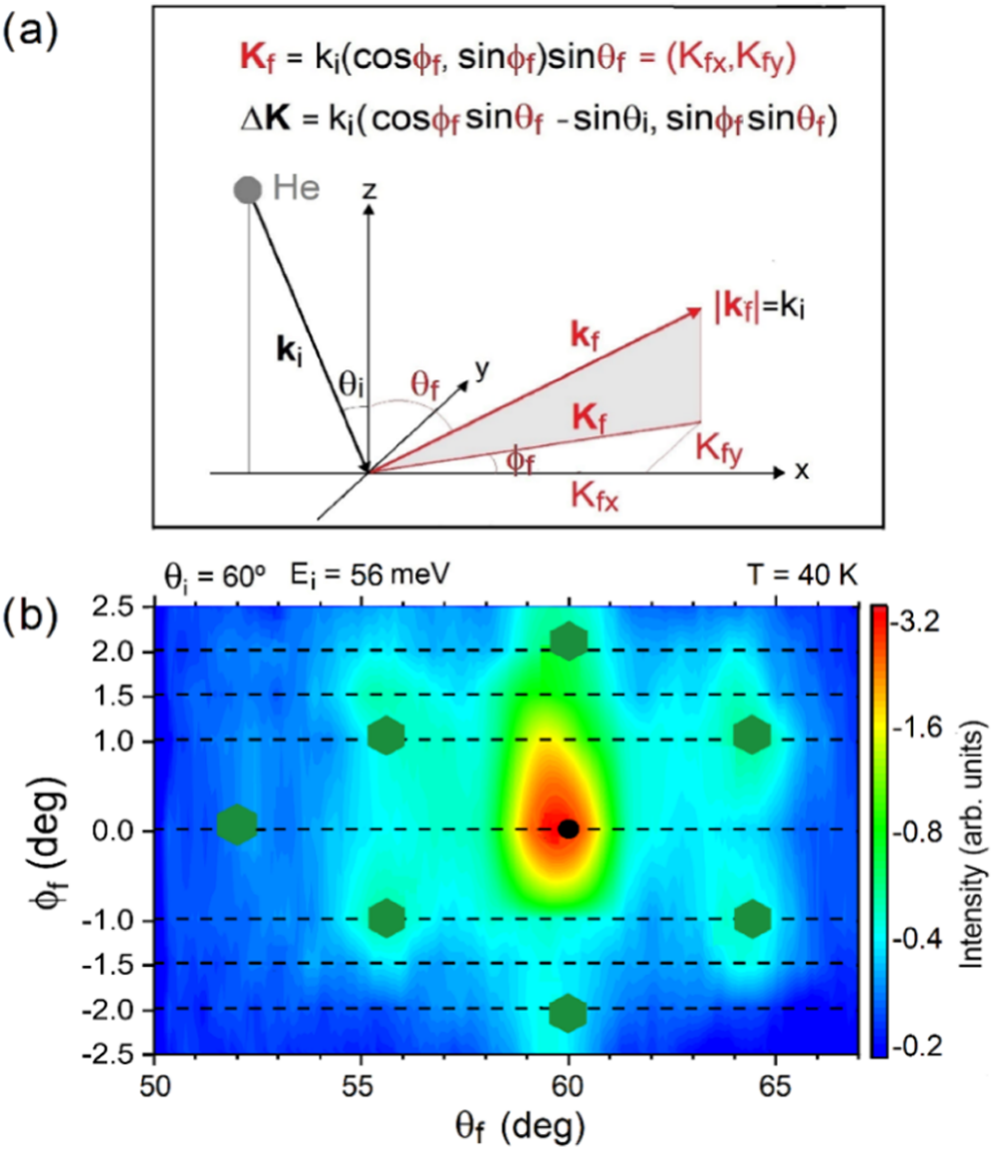
(a) Geometry and kinematics of out-of-plane HAS diffraction relating the components of the final parallel wavevector **K**_*f*_ and parallel wavevector transfer Δ**K** to the incident wavevector **k**_*i*_ (or incident energy through *k*_*i*_ [Å^–1^] = 1.383√*E*_*i*_[meV] for ^4^He atoms^8^) and scattering geometry. (b) The out-of-plane HAS diffraction map of single-layer NbSe_2_/BLG/SiC­(0001) at *T* = 40 K, measured as a function of the final and azimuthal angles defined in (a) at a fixed incident angle of 60° and energy of 52 meV. The 30°-rotated hexagonal pattern reflects the theoretical positions of the diffraction peaks (green hexagonal dots) for the (3√3 × 3√3)­30° superlattice.

It is worth mentioning that essentially the same diffraction spectra for the NbSe_2_ monolayer, including the out-of-plane map demonstrating the (3√3 × 3√3)­30° superlattice, have been measured at *T* = 40 K (see Figure SI-1), which indicates that there is no evidence for a (3 × 3) CDW pattern above 40 K. This is in agreement with previous STM results on the same system, which showed that a CDW sets in below 35 K,^3^ and in clear contrast with previous Raman measurements using samples exposed to ambient conditions where a critical temperature of 145 K has been reported.^4^

### 2.2 Debye–Waller Exponent

Figure 5 shows the temperature dependence of the Debye–Waller exponent, as derived from the ratio of He specular intensity at the surface temperature *T*, *I*(*T*) ≡ *I*, to that at the lowest measured temperature *T*_0_, *I*(*T*_0_) ≡ *I*_0_, for single-layer NbSe_2_ compared to the data for bulk NbSe_2_ and the few-layer NbSe_2_. The data for the NbSe_2_ overlayers are reported for two different He beam incident energies and angles.

**Figure 5:**
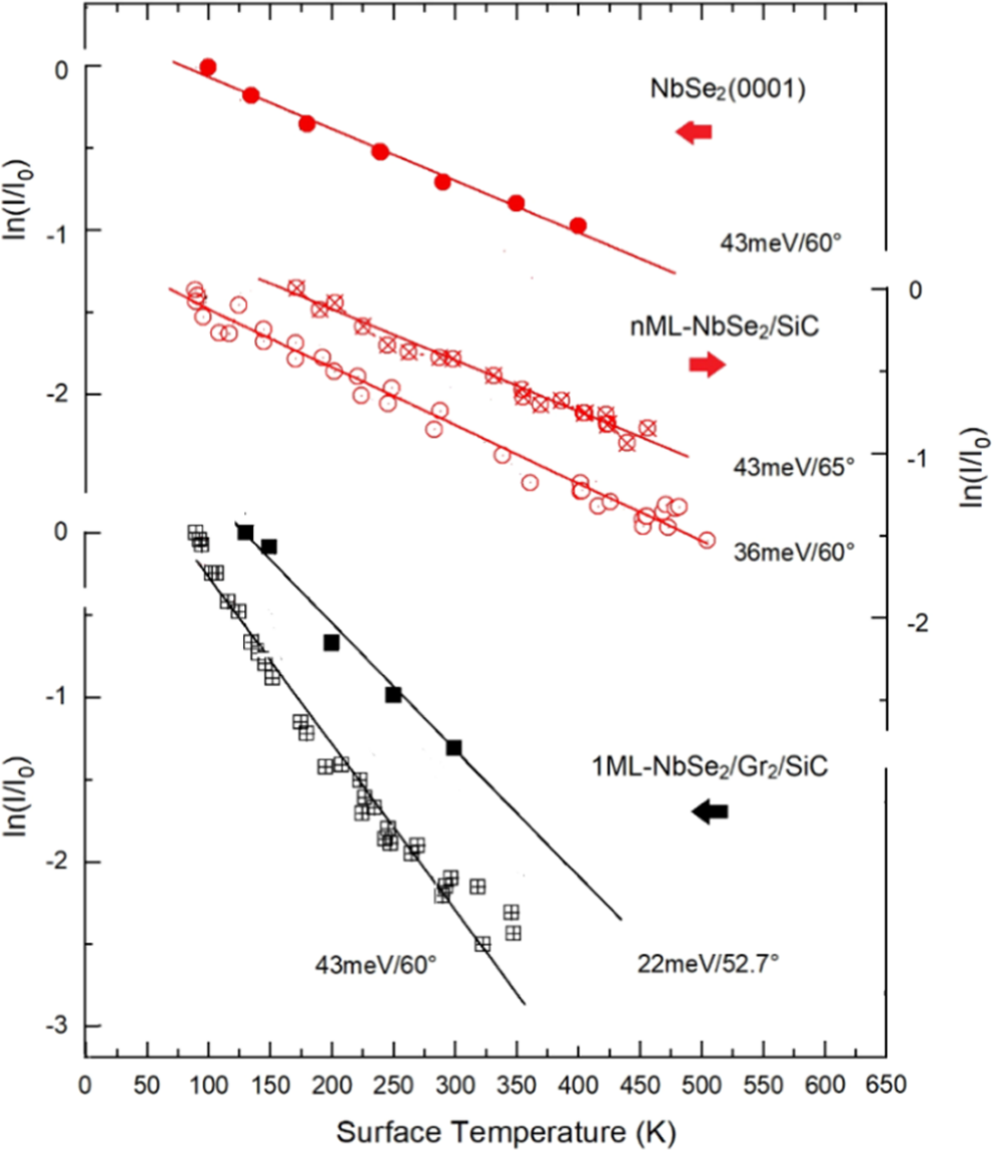
Thermal attenuation of He specular intensity measured along the ΓM azimuth from bulk NbSe_2_ (top), few-layer NbSe_2_/BLG/SiC­(0001) (middle), and for single-layer NbSe_2_/BLG/SiC­(0001) (bottom). Note the different ordinate scales for the three sets of data. The corresponding incident energy and incident angles are given for each set of data. The straight lines correspond to the best linear fits according to eq 2.

While the exponential attenuation of the specular intensity is about the same for the semi-infinite crystal and for the 2–3 layers, a much steeper attenuation is observed for the NbSe_2_ monolayer on top of the graphene bilayer. This is mainly due to the graphene substrate, which provides several intervalley channels, as discussed in previous HAS studies on the electron–phonon interaction of graphene on various substrates.^15^ In the next subsection, the corresponding values of the electron–phonon coupling constant are derived for the present set of data, based on the information from phonon dispersion curves derived from HAS TOF data. While HAS data for bulk NbSe_2_ phonons are already available^22^ and assumed to be approximately valid for multilayers, the HAS phonon data for the monolayer NbSe_2_ are presented in the following subsection.

### 2.3 Phonons

Two of the several sets of HAS TOF spectra of 1 ML-NbSe_2_/BLG/SiC­(0001), measured at a surface temperature of 100 K along the ΓM direction, are shown in Figure 6a,b as functions of the energy transfer Δ*E*_*i*_. The inelastic peaks in the TOF spectra, like those marked by small triangles in Figure 6a,b for phonon creation (Δ*E*_*i*_ *<* 0) and annihilation (Δ*E*_*i*_ *>* 0) processes, provide the phonon energy ℏω = |Δ*E*_*i*_| as a function of the parallel wavevector *Q* = |Δ*K*| (full black circles in Figure 6c). A possible set of dispersion curves is represented by the eye guidelines (gray full lines) drawn in Figure 6c. They are compared to the data for the 2H-NbSe_2_(0001) surface (red open symbols)^22^ and to the bulk NbSe_2_ dispersion curves (blue broken lines).^23^

**Figure 6:**
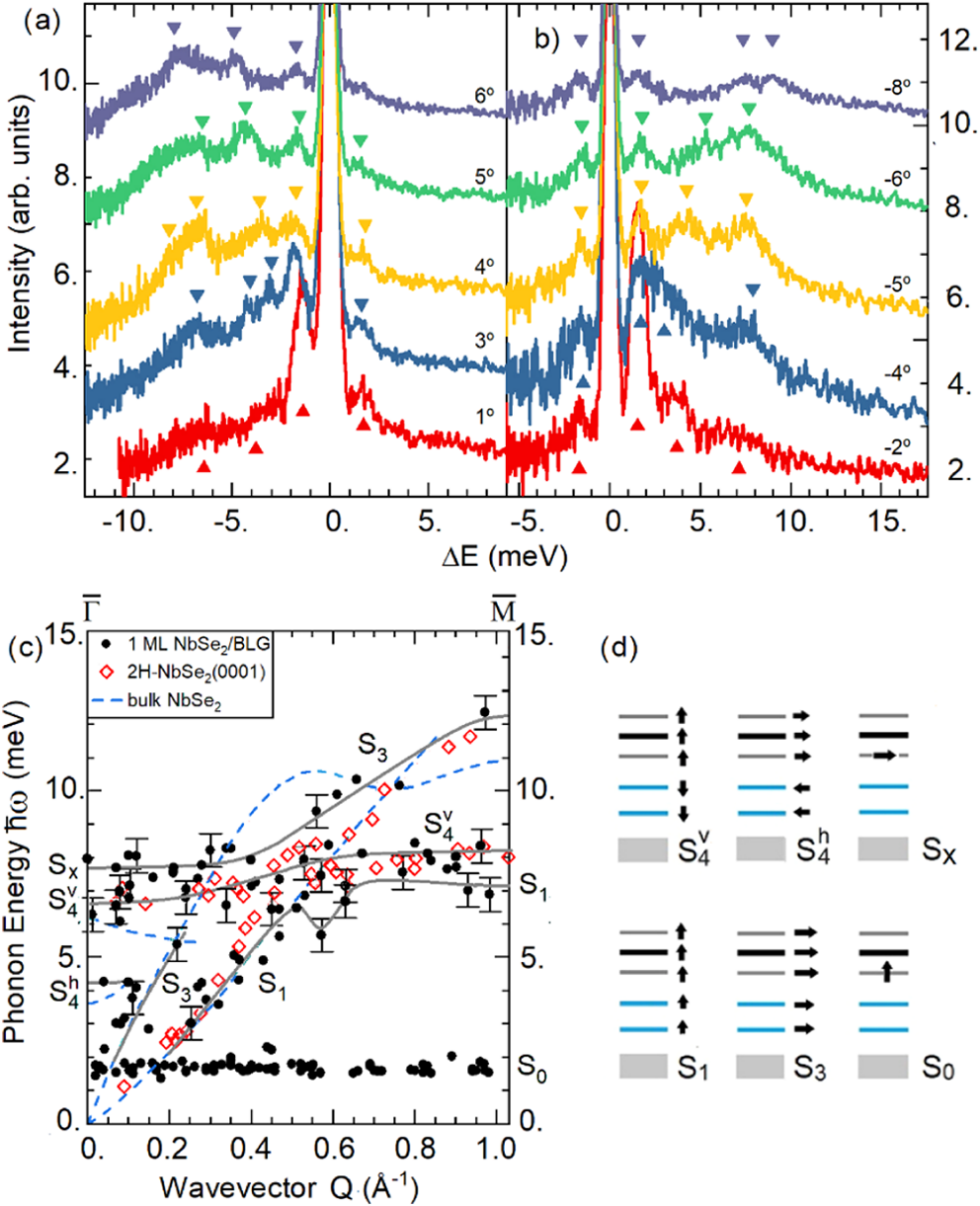
(a,b) Selected time-of-flight spectra measured with HAS at constant scattering angle θ_*i*_ + θ_*f*_ = 108°, incident energy *E*_*i*_ = 26 meV, along the ΓM direction of single-layer NbSe_2_/BLG at 100 K; each spectrum is labeled by the rocking angle φ of the surface plane, so that θ_*i*_ = 54° + φ and θ_*f*_ = 54° - *φ.* (c) The corresponding phonon dispersion curves (full black circles, with full gray lines as eye guidelines) are compared to those measured with HAS for the semi-infinite NbSe_2_(0001) surface at room temperature (red open symbol),^22^ and to the bulk NbSe_2_ dispersion curves (blue broken lines).^23^ Besides the surface branches S_1_, S_3_, S_4_^v^ and S_4_^h^, a flat soft branch S_0_ is observed in single-layer NbSe_2_/BLG at 1.7 meV, attributed to interface localized (Moiré) modes with vertical (z) polarization. The horizontal (x-polarized) counterpart is associated with the S_*x*_ branch at 8 meV, which converts via avoided crossing to the S_3_ branch at about ΓM/2. The displacement patterns of the atomic planes for the different branches are schematically shown in (d), with the BLG illustrated by blue segments, the NbSe_2_ three atomic layers by gray and black segments, and the SiC(0001) substrate, assumed to be rigid, by gray boxes.

In the long-wave limit the surface phonon branches in the acoustic region of 1 ML-NbSe_2_/BLG labeled as S_1_, S_3_, S_4_^v^ and S_4_^h^ correspond to the Rayleigh wave, the longitudinal resonance, the shear-vertical and longitudinal oscillations of the NbSe_2_ monolayer against the BLG (Figure 6d). In 2H-NbSe_2_(0001) the surface unit cell includes two NbSe_2_ monolayers, while in our system the BLG plays, to some extent, the role of the second monolayer. This may alone explain the softening of the S_1_ branch and the stiffening of S_3_, both in the second half of ΓM, with respect to those of bulk 2H-NbSe_2_(0001) (red open symbols), although the stiffening of S_3_ may also be related to the avoided crossing with S_*x*_. Note that a possible Kohn anomaly occurs in the S_1_ branch near 0.6 Å^–1^, more pronounced than that found with HAS at room temperature in bulk NbSe_2_(0001),^22^ and showing a shift to a smaller *Q* with respect to that reported with neutron scattering in bulk at ^2^/_3_ΓM (blue broken line).^23^ This is probably related to the charge transfer and the consequent shift of the Fermi wavevector. A similar shift of the anomaly has been observed with HAS in 2H-TaSe_2_(0001) for the Rayleigh mode with respect to the anomaly in the bulk acoustic mode.^24^

The low-energy flat phonon branch S_0_, displaying a strong intensity at small *Q* (Figure 6a.b) may be associated with soft shear-vertical vibrations localized at a superlattice periodic array of atoms, for example the interface Se ions which accommodate at the high symmetry hexagonal (and possibly trigonal) sites of graphene (corner atoms in Figure 3b) with strongly weakened local shear force constants. Note that this branch is reminiscent of the low energy dispersionless modes observed on Gr/Ir(111) and Gr/Ru(0001), where graphene builds a Moiré pattern.^25,26^ Due to this analogy and the present interpretation of the flat branch as closely associated with the interface superstructure, in the following discussion we shall term these localized excitations as *Moiré* phonons. On the other hand, such localized shear-vertical vibrations should have their longitudinal companion, which is here associated with the S_*x*_ additional branch, also localized above the maximum of the acoustic band in the region around the zone center.^27^ Clearly, only a surface dynamics first-principle calculation for this system would allow for a reliable assignment of the observed additional Moiré branches S_0_ and S_*x*_.

### 2.4 Electron–Phonon Interaction

The HAS data for ln­(*I*/*I*_0_), shown in Figure 5 for the surfaces studied, provide the HAS Debye–Waller (DW) exponent −2*W*(*T*), up to a constant that depends on how *I*_0_ is defined. Therefore, the slope of ln­(*I*/*I*_0_) as a function of temperature directly represents the slope of −2*W*(*T*), regardless of the specific definition of *I*_0_. This slope can be used to determine the electron–phonon coupling constant, λ_HAS_ (also known as the mass-enhancement factor), for bulk NbSe_2_ and the few layers. This approach is based on the method valid in the high-temperature limit.^12^

For single-layer NbSe_2_/BLG/SiC, at least two different contributions to λ_HAS_ are expected from phonons from the NbSe_2_ monolayer and Moiré phonons from the interface. The high-energy optical phonons of the BLG can be neglected, however, due to their small population in the present temperature range, and their depth beneath the first surface atomic layer. In this approximation, the expression of the DW exponent receiving contributions from two distinct phonon spectral regions can be written as in ref 28.

$$2W\left(k_{i},T\right)=\frac{a_{c}k_{\mathrm{iz}}^{2}}{\pi \phi }n_{s}\lambda_{\mathrm{HA}S}\left\{A\hbar \omega_{0}\;\cot h\left(\frac{\hbar \omega_{0}}{2k_{B}T}\right)+\left(1-A\right)\hbar \omega_{1}\;\cot h\left(\frac{\hbar \omega_{1}}{2k_{B}T}\right)\right\}$$ 1

where ω_0_ = 1.7 meV and ω_1_ = 13.5 meV are the frequency of Moiré phonons and the surface Debye frequency of bulk NbSe_2_ (taken as the bulk Debye frequency^29^ divided by √2)^8^, respectively, and the coefficient 0 ≤ *A* ≤ 1 weighs the two contributions. Although the Moiré phonons fall in the spectral region of acoustic phonons, their flat, optical-like dispersion, giving a sharp peak at ω_0_ in the phonon density, need to be treated separately from the acoustic phonons, which have instead a linear dispersion and are collectively represented by a surface Debye frequency ω_1_ ≫ ω_0_. In the prefactor of eq 1 r.h. member, *a*_*c*_ is the surface unit cell area, equal to 10.276 Å^2^ for the semi-infinite bulk NbSe_2_ ^30^ and for the few layers, while for single-layer NbSe_2_ the value *a*_c_ = 10.914 Å^2^ is determined from the measured lattice parameter including the dilation induced by charge transfer. Moreover, *k*_*iz*_ is the surface normal component of the incident He atom wavevector and ϕ the surface workfunction. For the semi-infinite NbSe_2_ the work function is ϕ = 5.9 eV.^7^ For the nML-NbSe_2_ film, the small reduction of the HAS diffraction intensities with respect to those of the semi-infinite NbSe_2_ (Figure 1) indicate a similarly small decrease of the surface corrugation due to a charge transfer from the SiC(0001) substrate, whose work function is ϕ = 4.6 eV.^6^ This value is used for the nML, while the even smaller workfunction of the substrate BLG/SiC(0001), ϕ = 4.3 eV,^6^ is used for the NbSe_2_ monolayer, which undergoes alone a massive charge transfer and therefore a flattening of the surface. Finally, the factor *n*_s_ in eq 1 represents the number of layers involved in the electron–phonon interaction probed by the scattering He atom. It stems from the experimental observation that for ultrathin conducting films the slope of *2W* as a function of *T* grows proportionally with the number *n* of layers and saturates to a constant *n*_*sat*_.^31^ This leads to the assumption that, as far as the DW exponent is concerned, a multilayer film may be viewed as a stack of 2D free-electron gases. In the present case, the nearly equal DW slopes in Figure 5 for nML (*n* = 2–3) and the semi-infinite NbSe_2_ indicate that also *n*_sat_ is in the range 2–3. In the following *n*_s_ = 2 shall be used for the semi-infinite and nML-NbSe_2_, consistently with the previous analysis of other 2H-stacked transition metal dichalcogenides (TMDs).^13,14,32^ The large increase of the DW slope observed for 1 ML/BLG/SiC(0001) is then attributed to the insertion of BLG. The model formulated above for the origin of Moiré phonons implies that also the graphene layer interfaced to the 1 ML NbSe_2_ has to contribute to the electron–phonon interaction via intra- as well as interpocket (Dirac cone) transitions. As discussed in,^15^ such a multivalley transition multiplicity contributes six units to the total *n*_s_, which is therefore *n*_s_ = 7. The second graphene layer acts as a buffer layer passivating the SiC(0001) surface^6^ and is assumed to play no role.

In the following analysis of the DW slopes based on eq 1, for a given value of *A*, the mass-enhancement factor λ_HAS_ works as a free parameter so as to obtain the best fit of the −2*W*(*T*) shape (equal to that of ln­(*I*(*T*)/*I*_0_)). In this way λ_HAS_ is obtained. The high-temperature limit is equivalent to letting ω_0_ and ω_1_ in eq 1 tend to zero, which gives

2 $$2W\left(k_{i},T\right)=\frac{2a_{c}k_{\mathrm{iz}}^{2}}{\pi \phi }n_{s}\lambda_{\mathrm{HAS}}k_{B}T$$

and therefore

3 $$\lambda_{HAS}=-\frac{\pi \phi }{2n_{s}a_{c}k_{\mathrm{iz}}^{2}}\frac{\partial \;\ln\left[I\left(T\right)/I_{0}\right]}{k_{B}\partial T}$$

as reported in ref 12.^12^

The best linear fits in the high-*T* limit (eq 2) for all the considered samples are shown in Figure 5 (straight lines) and give λ_HAS_ = 0.76 ± 0.06 for semi-infinite bulk NbSe_2_, λ_HAS_ = 0.82 ± 0.06 and 0.75 ± 0.06 for the 2–3 ML-NbSe_2_ data sets 43 meV/65° (*k*_*iz*_^2^ = 14.75 Å^–2^) and 36 meV/60° (*k*_*iz*_^2^ = 17.28 Å^–2^), respectively. For the NbSe_2_ monolayer on BLG/SiC the fit gives λ_HAS_ = 0.58 ± 0.04 or 0.63 ± 0.04 when measured with a 43 meV He beam at 60° incidence or with a 22 meV He beam at 52.7° incidence, respectively.

As discussed above in connection with eq 1, the λ_HAS_ for the two data sets of single-layer NbSe_2_ actually receives contributions from two distinct phonon spectral regions: Moiré phonons and the lattice dynamics of the NbSe_2_ layer. Figure 7 shows three fits based on eq 1 of the 43 meV/60° data, one with only the Moiré phonons contribution (*A* = 1), one with an equal share of the Moiré and the NbSe_2_ phonons (*A* = 0.5) and the third with only the NbSe_2_ monolayer phonons (*A* = 0). The two fits including NbSe_2_ phonons (*A =* 0.5, 0) yield both λ_HAS_ = 0.60 ± 0.04, which is consistent with the high-*T* limit for the 43 meV/60° data. All three fits adequately account quite well for the experimental slope above 150 K, where the high-*T* linearity is established. However, below this temperature the two fits for *A* ≠ 0 diverge from the one including only moiré phonons and from experiment. This suggests that the moiré phonons make a significant contribution to the electron–phonon interaction at low temperatures. In a sense, they serve as acoustic surface modes that exist in bulk NbSe_2_, but lose their character and localization at small Δ*K* in the film due to penetration into the much stiffer substrate. This specific role of the Moiré phonons, which preserve their localization also for *Q* → 0, is consistent with the observation that the Moiré phonons are detected by HAS in those regions of the parallel momentum transfer where they intersect the S_1_ and S_3_ branches.

**Figure 7:**
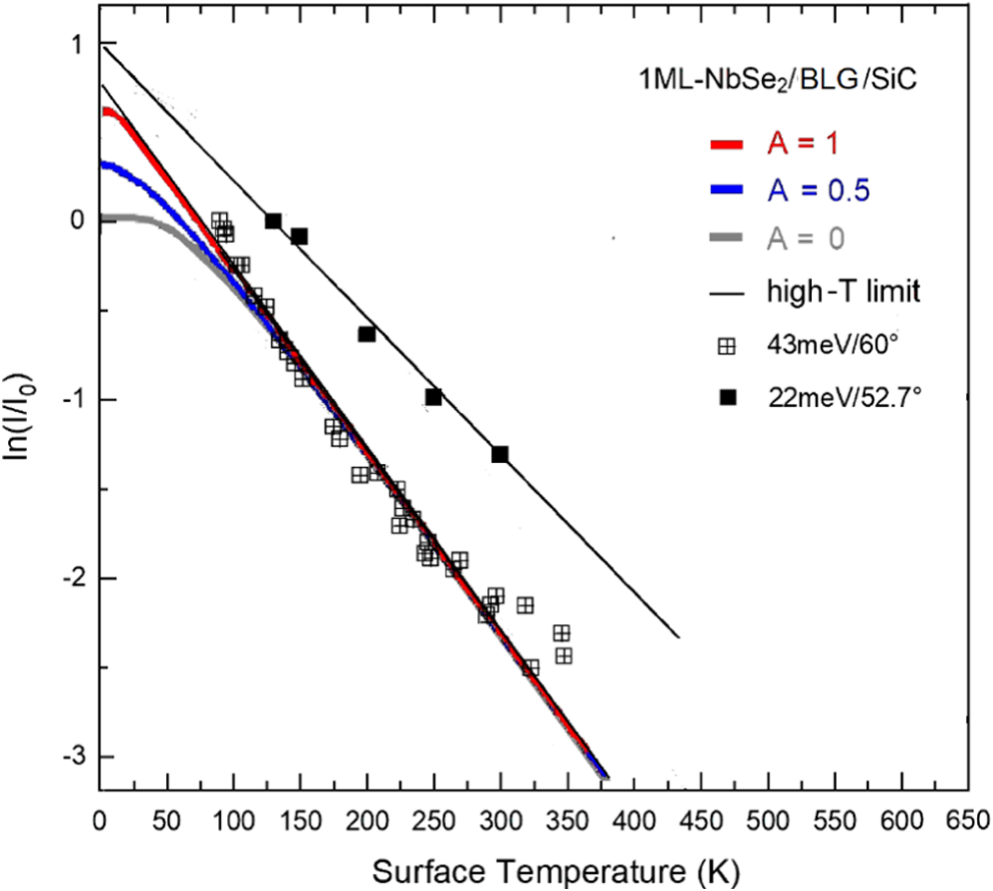
Fits of the Debye–Waller data for HAS incident energy *E*_*i*_ = 43 meV and angle θ_*i*_ = 60° using eq 1. The red curve represents a fit including only the Moiré phonons contribution (*A* = 1), the blue curve represents an equal share of the Moiré and the NbSe_2_ phonons (*A* = 0.5) and the gray curve includes only the NbSe_2_ ML phonons (*A* = 0). The few data for *E*_*i*_ = 22 meV and θ_*i*_ = 52.7°, all above 125 K, are well fitted by the high-*T* straight line, independently of *A*.

The above value of λ_HAS_ for the semi-infinite NbSe_2_ of 0.76 ± 0.06, compares well with the values obtained from photoemission measurements at low temperature by Valla et al. (0.85 ± 0.15 ^33^) and by Rahn et al. (in the range 0.7–0.9 ^34^). For the 2–3 ML the above values of λ_HAS_ = 0.82 ± 0.06 and 0.75 ± 0.06 turn out to be larger than that obtained by Lian et al. from first-principles for a Na-intercalated NbSe_2‑_bilayer (λ = 0.57 ^35^), where the Na atoms act as electron donors, like the SiC(0001) substrate in present experiments. A more instructing comparison is for the NbSe_2_ monolayer on BLG/SiC, whose average value of λ_HAS_ = 0.61 ± 0.04 is somewhat smaller than the values from first-principles calculations for a NbSe_2_ monolayer by Zheng and Feng (λ = 0.67),^36^ by Lian et al. for two different CDW configurations (λ = 0.84 and 1.09).^37^ It is also smaller than the λ ≈ 0.75 derived by Xi et al. from electrical transport and optical measurements for a NbSe_2_ monolayer on sapphire (work function 4.5 eV).^4^

### 2.5 Superconducting Critical Temperature

The superconducting critical temperature *Tc* can be derived from λ_HAS_ via the Allen–Dynes-modified McMillan formula^38,39^

4 $$T_{c}=11.5\frac{K}{meV}{\left\langle \omega \right\rangle }_{meV}\;\exp\left[-\frac{1.04\left(1+\lambda_{HAS}\right)}{\lambda_{HAS}-\mu^{*}-0.62\lambda_{HAS}\mu^{*}}\right]$$

with the Coulomb pseudopotential μ* = 0.15 and ⟨ω⟩_meV_ = 12.61 meV taken from.^40^ For the semi-infinite NbSe_2_ (λ_HAS_ = 0.76 ± 0.06) it is found *T*_c_ = 4.9 ± 0.6 K, which is smaller than the bulk value (7.3 K^41^). Comparable values of *T*_*c*_ are found from the two sets of data for few-layer NbSe_2_ (6.0 ± 0.7 K from λ_HAS_ = 0.82 ± 0.06 and 4.7 ± 0.6 K from λ_HAS_ = 0.75 ± 0.06) when the same values of μ* and ⟨ω⟩_meV_ are used for the NbSe_2_ multilayers. Encapsulated NbSe_2_ multilayers with 2 ≤ *n* ≤ 8 have shown a uniform decrease of *T*_c_ with thickness just in that range, while the *T*_c_ of the monolayer encapsulated with hBN drops to 2 K.^42^

For monolayer NbSe_2_ (curve labeled 43 meV/60° in Figure 7), the best-fit value λ_HAS_ = 0.58 ± 0.04, obtained by including the Moiré phonons (*A* = 1), yields, for ⟨ω⟩_meV_ = 12.61 meV, a smaller value of *T*_c_, equal to 1.84 ± 0.20 K. This compares well with previous experimental *T*_C_ values obtained for single-layer NbSe_2_/BLG/SiC using STM/STS (*T*_c_ = 1.5 K)^43^ and transport measurements *T*_c_ = 1.5 K.^44^ Despite the possible contributions of the Moiré phonons, the graphene interfacing has apparently the effect of depressing superconductivity.

## 3 Conclusions

High-resolution HAS diffraction data from single-layer NbSe_2_ on BLG/SiC(0001) reveal a (9 × 9)­0°, approximately reducible to a (3√3 × 3√3)­30° superstructure, commensurate with the underlying BLG lattice. Inelastic HAS data provide, besides a set of dispersion curves of acoustic and lower optical phonons, a soft, dispersionless branch of phonons at 1.7 meV, attributed to the interface localized defects distributed with the superstructure period, and thus termed Moiré phonons. The electron–phonon coupling for single-layer NbSe_2_/BLG has been derived from the temperature dependence of the DW exponent, and compared with those of few-layer NbSe_2_/BLG/SiC­(0001) and bulk NbSe_2_. The low-temperature behavior of the DW exponent suggests an appreciable contribution of the Moiré phonons to the electron–phonon coupling, although the intercalation of the BLG between the NbSe_2_ monolayer and the SiC substrate is seen to attenuate the electron–phonon coupling with respect to the case of a NbSe_2_ monolayer directly deposited on an inert substrate.

In conclusion, it has been demonstrated that high-resolution HAS diffraction studies can be advantageously extended to long-period conducting surfaces, e.g., those exhibiting long-period superstructures, in order to obtain a detailed structural information. Moreover, the electron–phonon interaction obtained from the analysis of the HAS-DW factor as a function of temperature proves to be, within the experimental uncertainties, quite reliable, also in the case of particularly complex superconducting 2D structures. The growing interest in twisted Moiré structures, realized through the assembling of 2D layered materials, should find in HAS a valuable tool for their structural and dynamical characterization.

## 4 Methods

### 4.1 Sample Preparation

Monolayers of NbSe_2_ were grown on epitaxial bilayer graphene (BLG) on 6H-SiC(0001) by molecular beam epitaxy at a base pressure of ∼3 × 10^–10^ mbar in our homemade UHV-MBE system at the DIPC in San Sebastián (Spain). SiC wafers with resistivities ρ ∼ 120 Ω cm were used. Graphitization of the SiC surface was carried out using an automatized cycling mechanism where the sample was ramped between 700 and 1350 °C at a continuous ramping speed of ∼20 °C/s. The SiC crystal was kept for 30 s at 1350 °C for 80 cycles.

Reflective high energy diffraction (RHEED) was used to monitor the layer growth of NbSe_2_. During the growth, the BLG/SiC substrate was kept at 570 °C. High purity Nb (99.99%) and Se (99.999%) were evaporated using an electron beam evaporator and a standard Knudsen cell, respectively. The Nb:Se flux ratio was kept at 1:30, while evaporating the Se led to a pressure of ∼4 × 10^–9^ mbar (Se atmosphere). Samples were prepared using an evaporation time of 30 min to obtain a coverage of ∼1–2 ML. To minimize the presence of atomic defects, evaporation of Se was subsequently kept for additional 5 min. Atomic Force Microscopy at ambient conditions was routinely used to optimize the morphology of the NbSe_2_ layers. The samples used for AFM characterization were not further used for STM.

Lastly, in order to transfer the samples from our MBE to Madrid for HAS measurements, they were capped with a ∼ 10 nm film of Se. The capping layer was easily removed in the UHV-HAS system by annealing the sample at ∼300 °C. The bulk NbSe_2_(0001) surface measurements have been performed after ex-situ exfoliation of a bulk NbSe_2_(0001) sample.

## Supplementary Material



## Notes

Additional experimental details and complementary results. It provides further details on the two HAS setups used in our experiments. HAS in-plane and out-of-plane diffraction spectra of the Moiré pattern formed by NbSe_2_ are presented in Figure S1. Figure S2 shows in-plane and out-of-plane HAS angular distributions measured along ΓM from the NbSe_2_ (0001) surface (PDF)

## References

[1] Naito M., Tanaka S. (1982). Electrical Transport Properties in 2H-NbS_2_, -NbSe_2_, -TaS_2_ and -TaSe_2_. J. Phys. Soc. Jpn..

[2] Foner S., McNiff E. J. (1973). Upper critical fields of layered superconducting NbSe2
at low temperature. Phys. Lett. A.

[3] Ugeda M. M., Bradley A. J., Zhang Y., Onishi S., Chen Y., Ruan W., Ojeda-Aristizabal C., Ryu H., Edmonds M. T., Tsai H. Z., Riss A., Mo S. K., Lee D., Zett A., Hussain Z., Shen Z. K., Crommie M. F. (2016). Characterization
of collective ground states in single-layer NbSe_2_. Nat. Phys..

[4] Xi X., Zhao L., Wang Z., Berger H., Forró L., Shan J., Mak K. F. (2015). Strongly
enhanced charge-density-wave
order in monolayer NbSe_2_. Nat. Nanotechnol..

[5] Semov W. I. (1969). Work Function
of Oxidized Metal Surfaces and Estimation of A1_2_0_3_ Film Band Structure Parameters. Phys. Status
Solidi.

[6] Mammadov S., Ristein J., Krone J., Raidel Ch., Wanke M., Wiesmann V., Speck F., Seyller T. (2017). Work function
of graphene
multilayers on SiC(0001). 2D Mater..

[7] Shimada T., Ohuchi F. S., Parkinson B. A. (1994). Work Function
and Photothreshold
of Layered Metal Dichalcogenides. Jpn. J. Appl.
Phys..

[8] Benedek, G. ; Toennies, J. P. Atomic-Scale Dynamics at Surfaces - Theory and Experimental Studies with Helium Atom Scattering; Springer-Nature, 2018.

[9] Li E., Hu J. X., Feng X., Zhou Z., An L., Law K. T., Wang N., Lin N. (2021). Lattice reconstruction
induced multiple ultra-flat bands in twisted bilayer WSe_2_. Nat. Commun..

[10] Lisi S., Lu X., Benschop T., de Jong T. A., Stepanov P., Duran J. R., Margot F., Cucchi I., Cappelli E., Hunter A., Tamai A., Kandyba V., Giampietri A., Barinov A., Jobst J., Stalman V., Leeuwenhoek M., Watanabe K., Taniguchi T., Rademaker L., van der Molen S. J., Allan M. P., Efetov D. K., Baumberger F. (2021). Observation
of flat bands in twisted bilayer graphene. Nat.
Phys..

[11] Arora H. S., Polski R., Zhang Y., Thomson A., Choi Y., Kim H., Lin Z., Wilson I. Z., Xu X., Chu J. H., Watanabe K., Taniguchi T., Alicea J., Nadj-Perge S. (2020). Superconductivity
in metallic twisted bilayer graphene stabilized by WSe_2_. Nature.

[12] Manson J. R., Benedek G., Miret-Artés S. (2022). Atom scattering
as a probe of the
surface electron-phonon interaction at conducting surfaces. Surf. Sci. Rep..

[13] Anemone G., Garnica M., Zappia M., Casado Aguilar P., Al Taleb A., Kuo C. N., Lue C. S., Politano A., Benedek G., Vázquez de Parga A. L., Miranda R., Farías D. (2020). Experimental determination of surface thermal expansion
and electron-phonon coupling constant of 1T-PtTe_2_. 2D Mater..

[14] Anemone G., Casado Aguilar P., Garnica M., Calleja F., Al Taleb A., Kuo C. N., Lue C. S., Politano A., Vázquez
de Parga A. L., Benedek G., Farías D., Miranda R. (2021). Electron-phonon coupling in superconducting 1T-PdTe_2_. npj 2D Mater. Appl..

[15] Benedek G., Manson J. R., Miret-Artés S. (2021). The Electron-Phonon
Coupling Constant
for Single-Layer Graphene on Metal Substrates Determined from He Atom
Scattering. Phys. Chem. Chem. Phys..

[16] Ribeiro-Palau R., Zhang C., Watanabe K., Taniguchi T., Hone J., Dean C. R. (2018). Twistable electronics
with dynamically
rotatable heterostructures. Science.

[17] Minniti M., Díaz C., Fernández Cuñado J. L., Politano A., Maccariello D., Martín F., Farías D., Miranda R. (2012). Helium, neon and argon
diffraction
from Ru(0001). J. Phys.: Condens. Matter.

[18] Zhu T., Litwin P. M., Rosul M. G., Jessup D., Akhanda M. S., Tonni F. F., Krylyuk S., Davydov A. V., Reinke P., McDonnell S. J., Zebarjadi M. (2022). Transport properties of few-layer
NbSe_2_: From electronic structure to thermoelectric properties. Mater. Today Phys..

[19] Yang G., Li L., Lee W. B., Ng M. C. (2018). Structure
of graphene and its disorders:
a review. Sci. Technol. Adv. Mater..

[20] McCann E., Koshino M. (2013). The electronic properties
of bilayer graphene. Rep. Prog. Phys..

[21] Barredo D., Laurent G., Nieto P., Farías D., Miranda R. (2010). High-resolution elastic and rotationally
inelastic
diffraction of D_2_ from NiAl(110). J. Chem. Phys..

[22] Benedek G., Manson J. R., Miret-Artés S., Ruckhofer A., Ernst W. E., Tamtögl A., Toennies J. P. (2020). Measuring the Electron-Phonon
Interaction in Two-Dimensional Superconductors with He Atom Scattering. Condens. Matter.

[23] Moncton D. E., Axe J. D., Di Salvo F. J. (1977). Neutron scattering study of the charge-density
wave transitions in 2H-TaSe 2 and 2H-NbSe 2. Phys. Rev. B.

[24] Benedek G., Brusdeylins G., Heimlich C., Miglio L., Skofronick J., Toennies J. P. (1988). Shifted Surface Phonon Anomaly in 2H-TaSe2(001). Phys. Rev. Lett..

[25] Al
Taleb A., Anemone G., Farías D., Miranda R. (2018). Resolving localized phonon modes on graphene/Ir(111)
by inelastic atom scattering. Carbon.

[26] Maccariello D., Campi D., Al Taleb A., Benedek G., Farías D., Bernasconi M., Miranda R. (2015). Low-energy excitations of graphene
on Ru(0001). Carbon.

[27] Weber F., Hott R., Heid R., Bohnen K. P., Rosenkranz S., Castellan J. P., Osborn R., Said A. H., Leu B. M., Reznik D. (2013). Optical phonons
and the soft mode in 2H-NbSe_2_. Phys.
Rev. B.

[28] Benedek G., Manson J. R., Miret-Artés S. (2022). The Role of
High-Energy Phonons in
Electron-Phonon Interaction at Conducting Surfaces with Helium-Atom
Scattering. Phys. Chem. Chem. Phys..

[29] Harper J. M. E., Geballe T. H., DiSalvo F. J. (1977). Thermal
properties of layered transition-metal
dichalcogenides at charge-density-wave transitions. Phys. Rev. B.

[30] Marezio M., Dernier P. D., Menth A., Hull Jr G. W. (1972). The crystal structure
of NbSe_2_ at 15 K. J. Solid State
Chem..

[31] Benedek G., Manson J. R., Miret-Artés S. (2020). The Electron–Phonon Interaction
of Low-Dimensional and Multi-Dimensional Materials from He Atom Scattering. Adv. Mater..

[32] Anemone G., Al Taleb A., Benedek G., Castellanos-Gomez A., Farías D. (2019). Electron-Phonon Coupling Constant
of MoS_2_. J. Phys. Chem. C.

[33] Valla T., Fedorov A. V., Johnson P. D., Glans P. A., McGuinness C., Smith K. E., Andrei E. Y., Berger H. (2004). Quasiparticle Spectra,
Charge-Density Waves, Superconduct-ivity, and Electron-Phonon Coupling
in 2H–NbSe_2_. Phys. Rev. Lett..

[34] Rahn D. J., Hellmann S., Kalläne M., Sohrt C., Kim T. K., Kipp L., Rossnagel K. (2012). Gaps and kinks
in the electronic
structure of the superconductor 2H-NbSe_2_ from angle-resolved
photoemission at 1 K. Phys. Rev. B.

[35] Lian C. S., Si C., Wu J., Duan W. (2017). First-principles study of Na-intercalated
bilayer NbSe2: Suppressed charge-density wave and strain-enhanced
superconductivity. Phys. Rev. B.

[36] Zheng F., Feng J. (2019). Electron-phonon coupling
and the coexistence of super-conductivity
and charge-density wave in monolayer NbSe_2_. Phys. Rev. B.

[37] Lian C. S., Si C., Duan W. (2018). Unveiling Charge-Density
Wave, Superconductivity, and
Their Competitive Nature in Two-Dimensional NbSe_2_. Nano Lett..

[38] McMillan W. L. (1968). Transition
Temperature of Strong-Coupled Superconductors. Phys. Rev..

[39] Dynes R. C. (1972). McMillan’s
equation and the Tc of superconductors. Solid
State Commun..

[40] Rossnagel K. (2011). On the origin
of charge-density waves in select layered transition-metal dichalcogenides. J. Phys.:Condens. Matter.

[41] Revolinsky E., Lautenschlager E. P., Armitage C. H. (1963). Layer structure superconductor. Solid State Commun..

[42] Cao Y., Mishchenko A., Yu G. L. (2015). Quality Heterostructures
from Two-Dimensional Crystals Unstable in Air by Their Assembly in
Inert Atmosphere. Nano Lett..

[43] Wan W., Dreher P., Muñoz-Segovia D., Harsh R., Guo H., Martínez-Galera A. J., Guinea F., de Juan F., Ugeda M. M. (2022). Observation of Superconducting
Collective Modes fromCompeting
Pairing Instabilities in Single-Layer NbSe_2_. Adv. Mater..

[44] Nakata Y., Sugawara K., Ichinokura S., Okada Y., Hitosugi T., Koretsune T., Ueno K., Hasegawa S., Takahashi T., Sato T. (2018). Anisotropic band splitting in monolayer NbSe_2_: implications
for superconductivity and charge density wave. npj 2D Mater. Appl..

