# Supplementary material for: Electron–Phonon Coupling and Phonon Dynamics in Single-Layer NbSe2 on Graphene: The Role of Moiré Phonons
Source: ACS Nano. 2025 Feb 26;19(9):8895–903. doi: 10.1021/acsnano.4c16399 (PMC12129249; doi:10.1021/acsnano.4c16399)
Supplement: Supplementary file 1 [file nn4c16399_si_001.pdf]

Supplementary Information

Electron-phonon coupling and phonon dynamics in single-layer $NbSe_2$ on graphene: the role of moiré phonons

Amjad Al Taleb[1], Wen Wan[2], Giorgio Benedek[2,3],
Miguel M. Ugeda[2,4,5] and Daniel Farías[1,6,7]

[1]Departamento de Física de la Materia Condensada, Universidad Autónoma de Madrid, 28049 Madrid, Spain
[2] Donostia International Physics Center, Paseo Manuel de Lardizábal 4, 20018 San Sebastián, Spain.
[3]Dipartimento di Scienza dei Materiali, Università di Milano-Bicocca, 20125 Milano, Italy
[4]Ikerbasque, Basque Foundation for Science, 48013 Bilbao, Spain.
[5]Centro de Física de Materiales, Paseo Manuel de Lardizábal 5, 20018 San Sebastián, Spain.
[6]Instituto Nicolás Cabrera, Universidad Autónoma de Madrid, 28049 Madrid, Spain
[7]Condensed Matter Physics Center (IFIMAC), Universidad Autónoma de Madrid, 28049 Madrid, Spain

Methods

HAS measurements

The single-layer $NbSe_2$ sample was mounted in the HAS chamber in Madrid and heated to 300ºC in ultra-high vacuum (UHV) for an hour to desorb the Se capping layer. After cooling down, the crystalline quality of samples was revealed by the observation in HAS of high specular intensity and diffraction features, like the ones shown in Fig. S1 below.

The samples were characterized by a set of HAS and time-of-flight measurements which were conducted in the Surface Science Laboratory in Universidad Autónoma de Madrid. The experiments have been carried out in two different systems, both having UHV chambers with base pressures in the low 10^{-10} mbar range. The first system [1] is a He-scattering apparatus in which the detector can rotate by 200º in the scattering plane, defined by the beam direction and the normal to the surface, and by +/-15º in the direction perpendicular to the scattering plane. This allows detection of both in-plane and out-of-

plane intensities for fixed incident conditions. The second chamber is a high-resolution He-scattering machine with a time-of-flight (TOF) arm and a fixed angle of 108º between the incident and outgoing beams [2]. In both HAS machines, the He atom beam is produced by a free expansion of helium gas through a 10 μm nozzle. The incident beam energy can be varied by changing the nozzle temperature. The sample can be heated by electronic bombardment or cooled with liquid nitrogen to 90K or liquid He (down to 40K). The sample can be moved in the X, Y and Z directions, rotated in the X-Y plane and changed the azimuthal angle (φ), which determines the incidence direction with respect to the lattice vector.

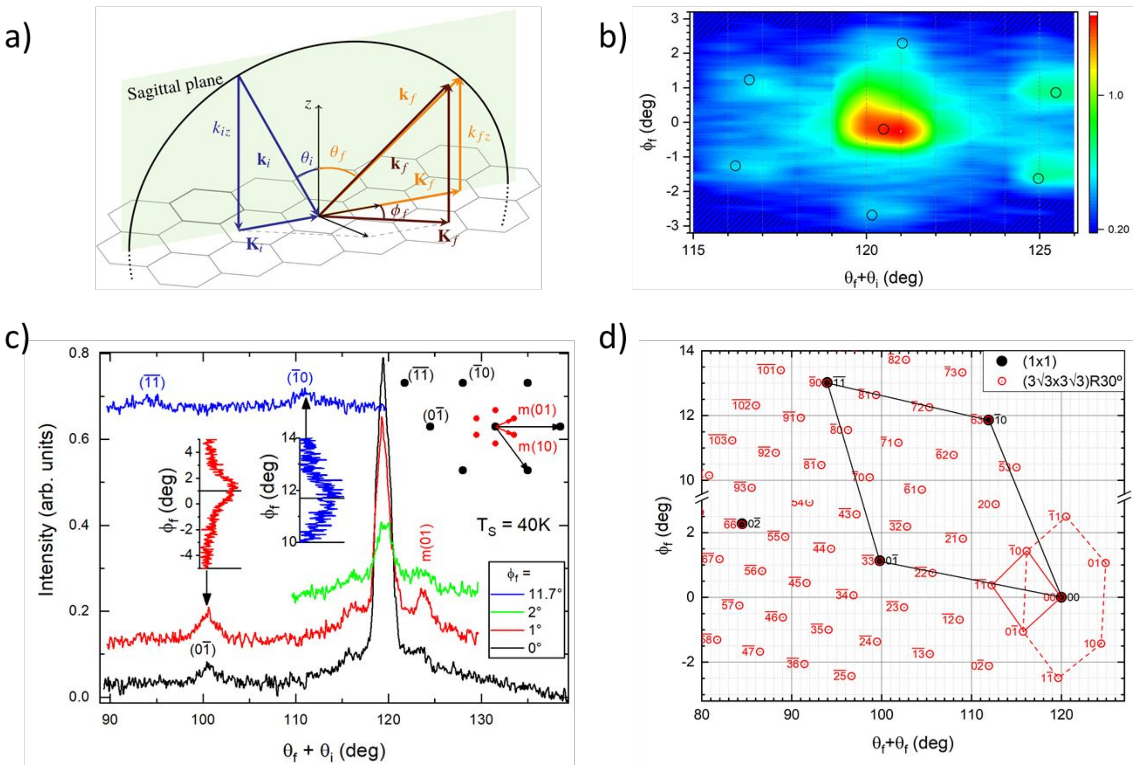

Figure S1. (a) Kinematics of elastic HAS in-plane and out-of-plane scattering, with $\mathbf{k}_i$ and $\mathbf{k}_f$ the incident and final He-atom wavevectors ($k_i = k_f$), θ_i and θ_f the incident and final polar angles, $\phi_i = 0$ and ϕ_f the incident and final azimuthal angles with respect to the ΓM direction of $NbSe_2$ (taken as the incident direction). $\mathbf{K}_i$ and $\mathbf{K}_f$ are the respective surface parallel components and $\Delta\mathbf{K} = \mathbf{K}_f - \mathbf{K}_i$. **(b)** 2D map of the moiré structure overserved on $NbSe_2$ with individual spectra collected

performing scans along ϕ_f. **(c)** HAS diffraction spectra collected along ϕ_f and θ_f shown as vertical and horizonal plots, respectively. Note that the maximum of the first order peak $(0\bar{1})$ appears at $\phi_f = 1^{\circ}$ (red vertical curve), due to azimuthal misalignment of the $NbSe_2$ lattice with respect to the sagittal plane $\phi_i = 0^{\circ}$. This offset can be traced back to a misalignment $\phi_i = 5^{\circ}$. **(d)** Calculated diffraction pattern of HAS from both the (1x1) $NbSe_2$ and (3√3x3√3)R30° surfaces under the experimental conditions: $E_i = 44$ meV, $\phi_i = 5^{\circ}$ and $\theta_i = 60^{\circ}$. The measurements shown in b) and c) were done with a sample temperature of 40K.

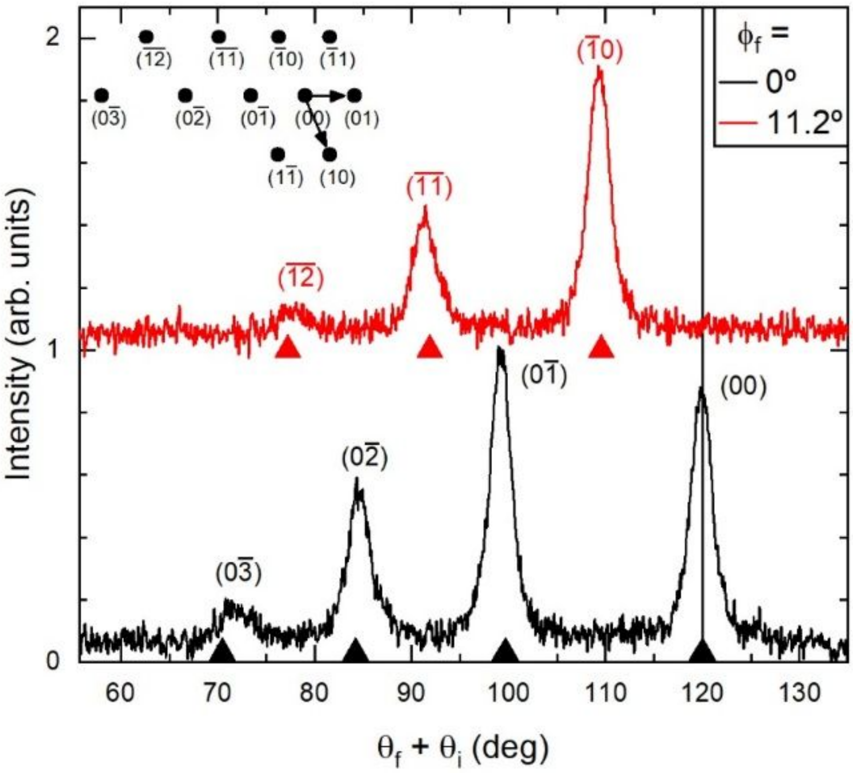

Figure S2. In-plane (black curve) and out-of-plane (red curve) angular distributions of HAS measured along ΓM from the surface of $NbSe_2$ (0001). The angle of incidence is 60° and $E_i = 44$ meV. Triangles indicate the expected position of diffraction peaks for a lattice constant a=3.44 Å and $\phi_i = 1.5^{\circ}$ due to azimuthal misalignment of the crystal.

References

[1] Minniti, M.; Díaz, C.; Fernández Cuñado, J.L.; Politano, A.; Maccariello, D.; Martín, F.; Farías, D.; Miranda, R. Helium, neon and argon diffraction from Ru(0001). *J. Phys.: Condens. Matter* **2012**, 24, 354002.

[2] Barredo, D.; Laurent, G.; Nieto, P.; Farías, D.; Miranda, R. High-resolution elastic and rotationally inelastic diffraction of D_2 from NiAl(110). *J. Chem. Phys.* **2010,** 133, 124702.
